# Hyperhomocysteinemia as an Early Predictor of Erectile Dysfunction: International Index of Erectile Function (IIEF) and Penile Doppler Ultrasound Correlation With Plasma Levels of Homocysteine

**DOI:** 10.1097/MD.0000000000001556

**Published:** 2015-10-02

**Authors:** Riccardo Giovannone, Gian Maria Busetto, Gabriele Antonini, Ottavio De Cobelli, Matteo Ferro, Stefano Tricarico, Francesco Del Giudice, Giulia Ragonesi, Simon L. Conti, Giuseppe Lucarelli, Vincenzo Gentile, Ettore De Berardinis

**Affiliations:** From the Department of Urology, Policlinico Umberto I Sapienza Rome University, Rome, Italy (RG, GMB, GA, ST, FDG, GR, VG, EDB); Department of Urology, European Oncology Institute (IEO), Milan, Italy (ODC, MF); Department of Urology, Stanford University of School of Medicine, Stanford, California, USA (SLC); and Department of Emergency and Organ Transplantation, Urology Unit, University of Bari, Bari, Italy (GL).

## Abstract

Erectile dysfunction (ED) is inability to achieve and maintain an erection to permit satisfactory sexual activity. Homocysteine (Hcys) is a sulfur-containing amino acid synthesized from the essential amino acid methionine. Experimental models have elucidated the role of hyperhomocysteinemia (HHcys) as a strong and independent predictor for atherosclerosis progression and impaired cavernosal perfusion.

The aim of this study is to investigate the serum levels of Hcys in our cohort of patients with ED, to compare these values with these of control population and to examine Hcys as a predictive marker for those patients who are beginning to complain mild–moderate ED.

A total of 431 patients were enrolled in the study. The whole cohort was asked to complete the International Index of Erectile Function (IIEF) questionnaire. The study population was divided in 3 main groups: Group A: 145 patients with no ED serving as a control group; Group B: 145 patients with mild or mild–moderate ED; Group C: 141 patients with moderate or severe ED. Each participant underwent blood analysis. All patients underwent baseline and dynamic penile Doppler ultrasonography.

We found in our cohort mean Hcys plasma concentrations significantly higher than the cut-off point in both groups B and C (18.6 ± 4.7 and 28.38 ± 7.8, respectively). Mean IIEF score was 27.9 ± 1.39, 19.5 ± 2.6, and 11.1 ± 2.5 for groups A, B, and C, respectively (*P* < 0.0001). In the penile Doppler ultrasonography studies, a high significant inverse correlation was detected between the mean values of the 10th minute's peak-systolic velocity (PSV) and Hcys levels for the groups B and C.

This establishes a dose-dependent association between Hcys and ED. Furthermore, we showed that Hcys was an earlier predictor of ED than Doppler studies, as the Hcys increase was present in patients with mild ED even before abnormal Doppler values.

## INTRODUCTION

Penile erection is a neurovascular phenomenon, which includes arterial dilatation, corporal smooth muscle relaxation, and corporal veno-occlusion.^1^ Erectile dysfunction (ED) is inability to achieve and maintain an erection to permit satisfactory sexual activity and may have a relevant impact on the quality of life of the patients and their partners.^2^

The incidence of ED increase with age and its prevalence is very high, affecting up to 53.4% of men aged 30 to 80 years.^3^ ED can be a manifestation of peripheral atherosclerosis and a potential early sign of coronary disease, with which it shares common risk factors such as obesity, smoking, dyslipidemia, and metabolic syndrome.^4^ Several pathological conditions that affect the blood vessels, such as atherosclerosis, can lead to vasculogenic ED through the important role that endothelial cells play in vascular homeostasis.^5^ Deregulated release of vasodilatation stimulating factors from endothelium is referred as to endothelial dysfunction (EDys) which can increase the risk of vascular events. Next to the traditional risk factors of age, BMI, and smoking, emerging risk factors have been proposed as predictors for cardiovascular disease as well as ED.^6^

Experimental models have elucidated the role of hyperhomocysteinemia (HHcys) as a strong and independent predictor for atherosclerosis progression and impaired cavernosal perfusion and these findings seemed to be confirmed by clinical trials performed on study population which assessed HHcys as a clinically relevant novel risk factor for ED.^7^

Homocysteine (Hcys) is a sulfur-containing amino acid synthesized from the essential amino acid methionine, introduced with diet.^8^ The main metabolic pathways of Hcys are: transulfuration that is activated after meals, in the presence of excess methionine, and remethylation, when there are low levels of methionine. In the latest pathway, Hcys is converted to methionine in a reaction catalyzed by the enzyme methionine synthetase (MS), whose cofactor is vitamin B12, and by the enzyme 5,10-methylenetetrahydrofolate reductase (MTHFR), for which folic acid (FA) is a cofactor.^9^

HHcys can depend on genetic defects, acquired conditions, or combination of both. Genetic defects cause a reduction in activity of the enzymes involved in the remethylation pathway of Hcys, such as MTHFR, MS. Causes of acquired conditions include vitamin deficiencies, particularly folates and vitamins B6 and B12, as well as renal impairment.^10^

Increased levels of Hcys and consequently decreased levels of FA were found to be able to inhibit NO-synthase, which is responsible of nitric oxide (NO) production, one of the most important vasodilatation mediator, whose deficiency plays an important role in the pathogenesis of EDys and ED.^11^

The aim of this study is to investigate the serum levels of Hcys in our cohort of patients with ED, to compare these values with a control population and to examine Hcys as a predictive marker for those patients who are beginning to complain mild–moderate ED.

## MATERIALS AND METHODS

Between January 2012 and November 2014, 359 patients affected by ED and 145 men serving as control group with an average age of 57.4 years (range: 41–70), were enrolled to this single-centre trial in order to assess the role of Hcys as a predictor and potential risk factor for ED.

All participants have been enrolled from our Andrology clinic at the Department of Gynecological-Obstetric Sciences and Urological Sciences, “Sapienza” Rome University.

In all patients, we evaluated body mass index (BMI) score, hypertension, smoking attitude, and physical activity.

Each participant underwent blood analysis, which included lipid panel (total cholesterol, LDL, HDL, triglycerides), fasting plasma glucose (FPG), Hcys, B12 vitamin, FA, total testosterone, LH, FSH, prolactine (PRL), and TSH. Venous blood samples were obtained in the morning after overnight fasting and were processed within 4 hours in order to prevent a temperature and time dependent increase in plasma Hcys.^12^ Blood values were obtained by using chemiluminescent and radioimmunoassays (RIA) methods.

In particular, plasmatic levels of Hcys were measured by using immunodiagnostic method (ELISA) and considering a reference range of 4.3 to 12 μmol/L. Levels >12 μmol/L were used as a cut-off point to assess the risk for the different groups.

Furthermore, all patients underwent baseline and dynamic penile Doppler ultrasonography using a 7.5–13 MHz high-frequency ultrasound probe. Patients were examined and the cavernous arteries were studied at baseline conditions after tactile stimulation and then following intracorporal injection of 5 to 20 μg of alprostadil.

Vascular flow parameters were registered at baseline and again at 5, 10, and 20 minutes postinjection. Patients were assessed with the penis aimed onto the abdomen and the probe was placed on the ventral penile surface.

Doppler examinations were performed by a single operator to minimize possible variability and normal response was defined by a peak-systolic velocity (PSV) more than 30 cm/second, end-diastolic velocity (EDV) under 3 cm/second and resistive index [RI = (PSV − EDV)/PSV] more than 0.8.

Diagnosis criteria for ED included arterial insufficiency (PSV < 30 cm/second, EDV ≤ 3 cm/second), veno-occlusive dysfunction (EDV > 3 cm/second, PSV ≥ 30 cm/second, RI < 0.8), and mixed vascular disorder (PSV < 30 cm/second, EDV > 3 cm/second, RI < 0.8).^13^

The whole cohort was asked to complete the International Index of Erectile Function (IIEF) questionnaire that is the self-administered IIEF based on 15 questions to evaluate ED. We explored questions 1, 2, 3, 4, 5 about erectile function and question 15 on personal confidence with a total score in the range between 6 and 30.

The exclusion criteria were diabetes and metabolic syndrome, coronary artery disease, stroke, and neurological diseases (Parkinson's disease, Alzheimer disease, amyotrophic lateral sclerosis, etc.), major psychiatric disorders, hypogonadism, and other hormonal diseases (measured through total serum testosterone, LH, PRL, and TSH).

We have also excluded patients with a history of alcoholism and consumers of illicit drugs, because these may lead to a chronic reduction of folate levels and vitamins of Group B. Taking into account the aforementioned criteria, 73 patients were excluded.

Therefore, according to our criteria, the study population was divided in 3 main groups. In Group A, 145 patients with no ED (score ≥26) were included and used as a control group, in Group B, further 145 patients with mild or mild–moderate ED (score ≥17 and <26) and in Group C 141 patients with moderate or severe ED (score <17).^14^

All the patients from the control group were healthy men who voluntarily decided to participate to the study.

The Ethical Committee of Department of Gynecological-Obstetric Sciences and Urological Sciences, “Sapienza” Rome University approved the study protocol. The study was conducted in line with European Urology and Good Clinical Practice guidelines, with ethical principles laid down in the latest version of the Declaration of Helsinki. Every patient signed an informed consent to participate in the study.

Statistical calculations were performed with MedCalc 9.2.0.1 (MedCalc Software, Ostend, Belgium). Hcys plasma levels were compared between groups by the Mann–Whitney *U* test. Descriptive analysis was based on mean values with range in case of quantitative variables and on frequencies and percentages in case of ordinal and categorical factors. Differences between groups were accordingly based on Kruskall–Wallis test or the Chi-square test.

The Spearman correlation was used to evaluate the association of Hcys levels with IIEF domain scores and PSVs. Logistic regression was used to evaluate the combined predictive effect of the risk factors for severe ED. We performed a backward selection procedure with a removal criterion of *P* > 0.10 based on the likelihood ratio test. Model calibration was measured by the Hosmer–Lemeshow goodness of fit test. Statistical significance was defined as *P* ≤ 0.05.

## RESULTS

Clinical data and fasting laboratory findings detected in the 3 different groups are summarized in Table 1. Patients with ED in Groups B and C were on average older when compared to the control group (57.8 ± 4.1 and 58.9 ± 7.2 vs. 55.3 ± 3.5).

**TABLE 1:**
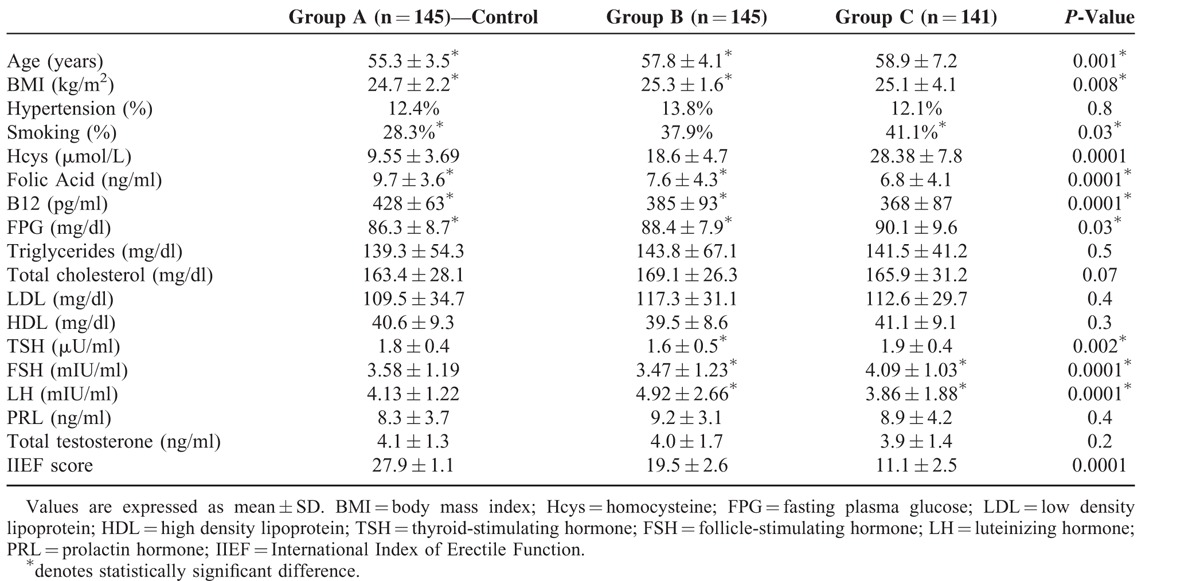
Participant Clinical Data and Laboratory Findings

The patients complaining any grade of ED were likely to be active smokers (37.8% and 41.2% for Groups B and C, respectively, vs. 28.3% for Group A).

Statistically significant differences were detected in BMI, serum levels of FPG, TSH, FSH, and LH while no significant differences were detected in prevalence of hypertension, triglycerides, total cholesterol, LDL, HDL, PRL, and total testosterone.

**FIGURE 1:**
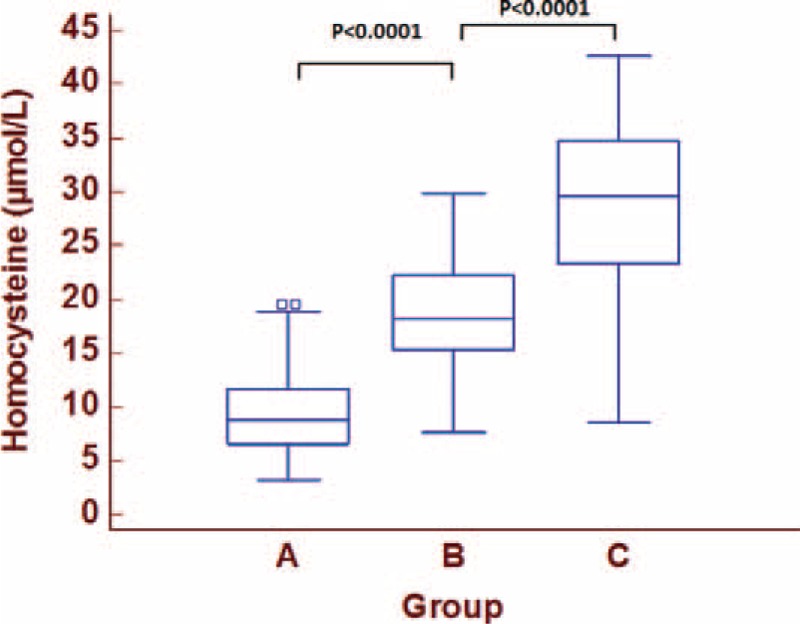
Differences in plasma concentrations of Homocysteine among the 3 groups.

Mean plasma levels of Hcys were significantly different in the cohort showing increasing values in the groups and the highest levels in patients with severe ED (Figure 1).

With a reference range between 4.3 and 12 μmol/L, we found in our cohort mean Hcys plasma concentrations steady higher than the cut-off point in both groups B and C (18.6 ± 4.7 and 28.38 ± 7.8, respectively).

Mean IIEF score was 27.9 ± 1.39, 19.5 ± 2.6, and 11.1 ± 2.5 for Groups A, B, and C, respectively (*P* < 0.0001).

**FIGURE 2:**
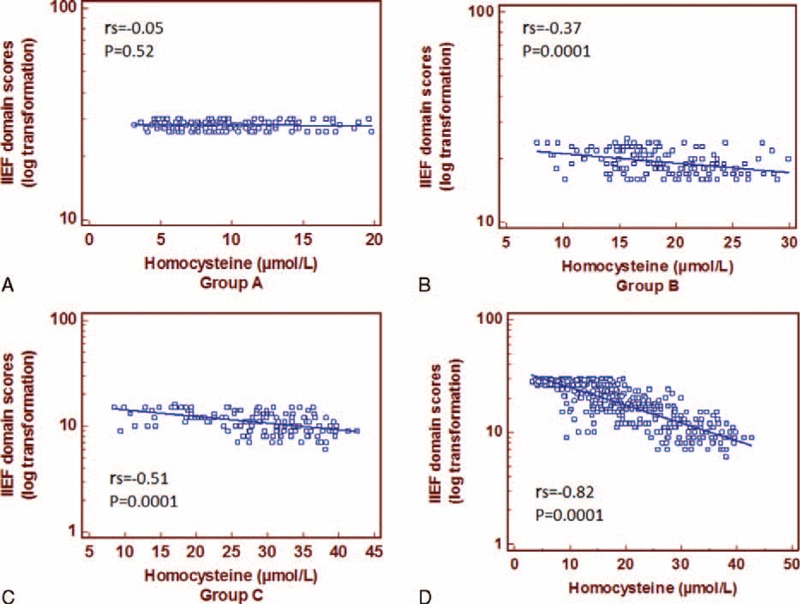
Spearman correlation coefficient (rs) and linear regression line between IIEF domain scores and homocysteine plasma levels in Groups A, B, and C, and overall population.

Mean plasma Hcys levels were negatively correlated with IIEF domain scores for both Groups B (rs = −0.37; *P* = 0.0001) and C (rs = −0.51; *P* = 0.0001), while in the control group we found no significant correlation (rs = −0.05; *P* = 0.52) (Figure 2).

**FIGURE 3:**
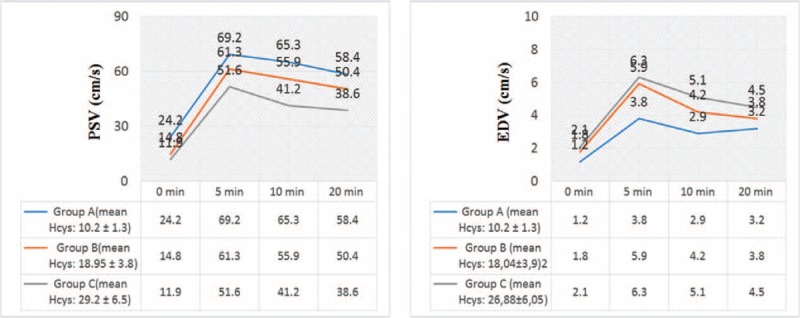
(A) Mean peak systolic velocity (cm/second) detected in the 3 different groups at 0′, 5′, 10′, and 20′ in penile Doppler ultrasonography; (B) mean end diastolic velocity (cm/second) detected in the 3 different groups at 0′, 5′, 10′, and 20′ in penile Doppler ultrasonography.

Penile Doppler ultrasonography results detected in the 3 groups are summarized in Table 2. A relevant gap between the PSV values in all groups was found (69.2 vs. 61.3 vs. 51.6 at 5 minutes; 65.3 vs. 55.9 vs. 41.2 at 10 minutes; 58.4 vs. 50.4 vs. 38.6 at 20 minutes; Figure 3A) and also between EDV parameters (3.8 vs. 5.9 vs. 6.3 at 5 minutes; 2.9 vs. 4.2 vs. 5.1 at 10 minutes; 3.2 vs. 3.8 vs. 4.5 at 20 minutes; Figure 3B).

**TABLE 2:**
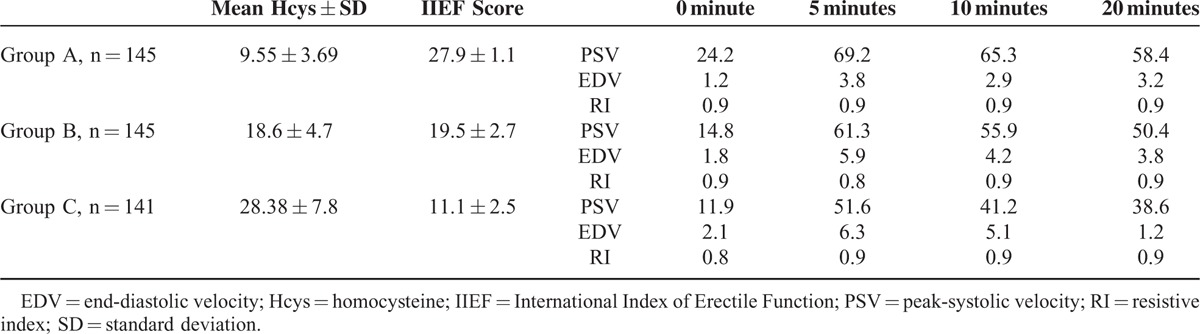
Penile Doppler Ultrasonography Results in Groups A, B, and C

**FIGURE 4:**
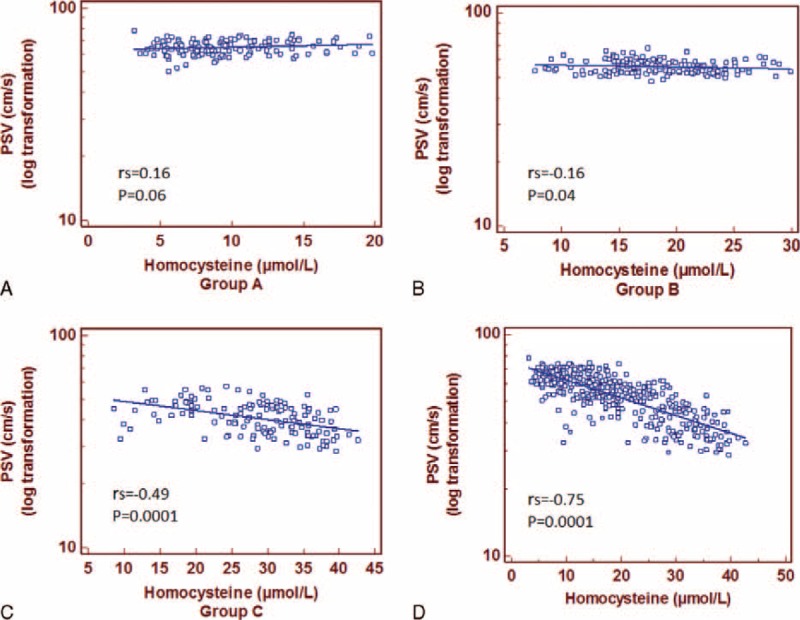
Spearman correlation coefficient (rs) and linear regression line between peak systolic value (PSV) and homocysteine plasma levels in Groups A, B, and C, and overall population.

A high significant inverse correlation was detected between the mean values of the 10th minute's PSV and Hcys levels for the Group B (rs = −0.16; *P* = 0.04) and C (rs = −0.49; *P* = 0.0001) (Figure 4).

By logistic regression considering age, BMI, arterial hypertension, smoking, and Hcys as independent variables, the only significant variables were age (OR = 1.03, 95% CI: 1.01–1.15; *P* = 0.0001) and Hcys levels (OR = 0.86, 95% CI: 1.23–1.35; *P* = 0.0001). The Hosmer–Lemeshow statistic was 5.2 (*P* = 0.4), thus, showing no evidence of poor calibration of the logistic regression model.

## DISCUSSION

ED, the second most common sexual disorder after premature ejaculation, has a significant impact on the quality of life of men and their partners^2^ and its incidence increase with age and is connected with several comorbidities.^3^

Vascular, hormonal, lifestyle, aging, neurologic, and psychological factors all play a role in the etiology of ED.^15^ Several associated metabolic imbalance contribute to the pathogenesis of EDys through a decreased vascular NO bioavailability, impaired vasodilatation, enhanced inflammation, and oxidative stress with proliferation of smooth muscle cells.^16^ Cavernous endothelial cell dysfunction is considered one of the most important pathological change in vasculogenic ED.^17^

Hcys has been known as an independent risk factor for vascular disease since 1999, when “The Rotterdam Study” evaluated the association of myocardial infarction and stroke with elevated levels of Hcys. Patients with HHcys had an odds ratio of 2.43 and 2.53 for myocardial infarction and stroke, respectively.^18^ Similar findings were obtained by Rasouli et al who indicated that the presence of increased Hcys levels (>12 μmol/L) strongly and independently predict progression of coronary plaque burden.^8^

Based on the association of Hcys with cardiac and vascular events, HHcys was introduced as possible mechanism in the pathogenesis of ED.

Khan et al showed the correlation between EDys and ED in experimental models in 1999. This work studied the contribution of free radicals production on ED in the corpora cavernosum of rabbits. This study highlighted how HHcys was able to induce an impaired cavernosal smooth muscle relaxation due to his capacity to inhibit NO-synthase.^19^

Elevated levels of Hcys can be reasonably included in a group of emerging risk factors involved in the pathogenesis of EDys that can ubiquitously affect the vascular system.

Hcys is a precursor of hydrogen sulfide (H_2_S), a vasodilator that has an anti-inflammatory, anti-apoptotic, and anti-oxidant properties.^20^

The role of Hcys in the EDys is due to a decrease of H_2_S, a novel mediator in cardiovascular homeostasis, and also to a decrease of NO synthesis through an increase reactive oxygen species (ROS) levels.^21^

In 2006, Demir et al studied the role of elevated plasma levels of Hcys in a cohort of 31 nondiabetic patients; this experience represented the first trial who detected on a study-based population, a significant association between HHcys and ED. In particular, the chance of developing ED was associated for the 80% in the cut-off point of patients who presented plasmatic levels of Hcys greater than 12.1 μmol/L; moreover, in those patients with HHcys was statistically showed a significant association with impaired flow-mediated vasodilatation and vascular abnormalities detected via penile Doppler ultrasound.^7^

In another study confirming the role of HHcys as a risk factor for ED, Lombardo et al showed that the mutation of the MTHFR gene is a genetic condition that can independently lead to HHcys. In this cohort of patients, all the participants were treated with vitamin B6 and FA therapy revealing an interesting improvement in the IIEF domain scores after the treatment as well as a significant improvement between the values of Hcys at baseline and at the end of the study.^9^

As already reported in literature,^7^ our study provides additional evidence that Hcys levels are elevated in the group of patients who complained a severe grade of disease (IIEF score < 17). A biochemical association between the Hcys levels and ED was also revealed by penile Doppler ultrasound studies, which demonstrated the severity of the disease in patients of Group C. More interesting, our experience revealed a moderate but steady increasing of the levels of Hcys in those patients (Group B) who were beginning to complain a mild or mild–moderate ED (IIEF score ≥17 and <26). This establishes a dose dependent association between Hcys and ED. Another important aspect of our study is that we showed that Hcys was an earlier predictor of ED than Doppler studies, as the Hcys elevation was present in patients with mild ED before abnormal Doppler values. This is remarkable in Group B where, even with normal Doppler ultrasound examination, Hcys is already elevated and higher than 12 μmol/L, considered as maximum reference level.

The main limitations of this study include the single-center nature of the report that may have an impact on its external validation, and the single measurement of the Hcys. A series of comprehensive blinded validation studies are warranted to confirm the utility of this biomarker in the clinical setting.

In conclusion, Hcys plasma levels could potentially be monitored as an early marker of ED prediction and could be used to identify those patients who may go on to develop severe ED. According to these findings, Hcys could be proposed as a novel biomarker in the diagnosis of ED and the correction of Hcys plasma levels, may represent a future target for monitoring therapy.

## CONCLUSIONS

HHcys correlates with ED onset and its plasma levels correlate with ED grade of severity. Considering our trial results, in a next future, Hcys could be used as an early predictor of ED development.
